# *Nocardia caishijiensis* infection: a case report and review of the literature

**DOI:** 10.1186/s12879-023-08186-z

**Published:** 2023-04-06

**Authors:** Fabricio Malaguez Webber, Arun Nachiappan, Freddy Duarte Lau, Christie Costello, Saul Zane

**Affiliations:** 1 Department of Internal Medicine, Bridgeport Hospital/Yale New Haven Health, Bridgeport, CT USA; 2 Department of Pharmacy, Bridgeport Hospital/Yale New Haven Health, Bridgeport, CT USA

**Keywords:** *Nocardia caishijiensis*, Pulmonary nocardiosis, Cavitary pneumonia

## Abstract

**Background:**

*Nocardia caishijiensis* is a rare soil actinomycete first described in Anhui province, China, in 2003. There has been only one reported instance of human infection caused by this species in the current literature.

**Case presentation:**

We present a case of pulmonary nocardiosis caused by *Nocardia caishijiensis* in a fifty-two-year-old man with human immunodeficiency virus infection and concomitant use of high-dose dexamethasone for cervical myelopathy, treated successfully with amikacin and thrimetroprim-sulfametoxazole, antibiotic resistance pattern was obtained, although interpretation may be limited.

**Conclusion:**

To our knowledge, this is the first reported case of *Nocardia caishijiensis* infection in humans in North America and the second one in the literature, this pathogen should be recognized as a potentially rising etiology of nocardiosis, especially in solid organ transplant recipients. This has a rising importance as the survival for solid organ recipients continue to rise with advance in transplant medicine leading to increased life expectancy in this particularly susceptible group.

**Supplementary Information:**

The online version contains supplementary material available at 10.1186/s12879-023-08186-z.

## Background

*Nocardia* is a genus of aerobic, variably acid-fast, gram-positive actinomycetes found in soil and water worldwide [1, 2]. Human infection is most often a result of direct inoculation of the skin or inhalation, especially in the context of immunosuppression. Common sites of *Nocardia* infection include pulmonary, cutaneous or central nervous system, with disseminated infection involving multiple organ systems a less common but potentially life-threatening manifestation. Recent reports suggest that the incidence of nocardiosis is increasing in line with increasing numbers of elderly and immunosuppressed patients [2, 3]. Over approximately one-hundred *Nocardia* species have been described, with over fifty of these involved in human disease. Shifts in speciation methods from biochemical to molecular genetic techniques has resulted in significant changes to our understanding of *Nocardia* taxonomy, with a recent increase in the number of recognized *Nocardia* species [4]. Identification of *Nocardia* species is clinically important as antibiotic susceptibility has been found to vary between species [5–7]. Since its original description in 2003 [8], *Nocardia caishijiensis* has not been reported to cause infection in humans in North America. Here, we present a case of pulmonary nocardiosis as the first documented case of human infection by *N. caishijiensis* in North America and the second one in the literature.

## Case presentation

A fifty-two-year-old male with a diagnosis of known human immunodeficiency virus infection presented to the emergency department with a four-day history of cough productive of grapefruit-colored sputum, hemoptysis and pleuritic chest discomfort, as well as a three-week history of unintentional twenty-pound weight loss. He denied fever, chills, rigors, night sweats, and dyspnea. Of note, he had been started on dexamethasone three weeks before admission after he was found to have worsening weakness of the extremities due to cervical myelopathy secondary to intervertebral disc herniation.

His past medical history was significant for chronic obstructive pulmonary disease, recently-diagnosed diabetes mellitus type 2, and hypertension. Of note, he ran out of and did not take his anti-retroviral therapy for three weeks a few months before admission (CD4 count six months before admission was 664 cells per microliter, and viral load two months before admission was 3.08 log copies per milliliter) although he had then resumed a couple of weeks leading to his presentation. His medication history was notable for abacavir-dolutegravir-lamivudine, amlodipine, aspirin, metformin, citalopram, dexamethasone, nicotine patches, albuterol and tiotropium inhalers; with no other regular or over-the-counter medications, or alternative remedies; and with no known drug allergies. His family history was non-contributory. His social history was significant for residency in a home and employment in sanitation and wall-painting; minimal alcohol use; minimal tobacco use at the time of admission, but with a fifty-pack-year history; and no illicit drug use, though he has a remote history of crack cocaine use. Of note, the patient does not endorse a history of exposure to sick contacts, international travel, or incarceration. His review of systems was otherwise unremarkable.

On examination, he was afebrile, with otherwise normal vital signs, with decreased breath sounds bilaterally but no crepitations, increased fremitus, or pectoriloquy; and an otherwise unremarkable examination. His initial laboratory studies revealed a white blood cell count of 10.2 × 1000/µL with left shift and an absolute neutrophil count of 8.6 × 1000/µL [2.2–7.2 × 1000/µL], a normal absolute lymphocyte count, a mildly elevated lactate dehydrogenase, a normal procalcitonin, and a blood glucose value of 422 mg/dL. His chest radiograph was remarkable for two opacities in the left middle zone with surrounding areas of consolidation. A subsequent CT chest showed the presence of cavitary lesions on the lingula, left upper and lower lobes, and right upper lobe. These opacities were in close proximity to the peripheral vasculature. Of note, these findings were not present on a prior CT chest performed seven months prior to admission for surveillance of a stable pulmonary nodule. He had blood and sputum cultures sent and was subsequently started on ceftriaxone and azithromycin. He underwent additional studies including legionella and streptococcal urine antigen, MRSA nasal swab, serial acid-fast bacilli stains of sputum, serum aspergillosis galactomannan, and 1,3-ß-d-glucan – all of which were negative. His repeat CD4 count was 119 cells/µL and his HIV viral load was undetectable. He was started on trimethoprim-sulfamethoxazole (TMP-SMX) for *Pneumocystis jirovecii* pneumonia (PJP) prophylaxis. His sputum culture was positive for *Pseudomonas oryzihabitans*, and an unspecified Nocardial species. He was started on amikacin, and therapeutic dose TMP-SMX, with discontinuation of other antibiotics. He subsequently underwent bronchoscopy with bronchoalveolar lavage (BAL) and transbronchial biopsy of the lingula and left lower lobe. He underwent an MRI of the brain, which was unremarkable. Given his stable power and sensation, his dexamethasone was tapered. Gram stain of the BAL fluid demonstrated 1 + white blood cells, 1 + gram-positive cocci, and 1 + gram variable rods, and cultures grew heaped, irregular, dry white “molar-shaped” colonies on blood agar that yielded beaded gram-positive rods upon staining. The isolate recovered was subjected to 16S rRNA sequencing using the MicroSeq 500 16S rDNA Sequencing Kit (ThermoFisher Scientific). The generated sequence was analyzed using the NCBI Blast database and was identified as *Nocardia caishijiensis.*

Antibiotic susceptibility testing was performed by Quest Diagnostics Infectious Disease, using broth microdilution trays. After organism inoculation and incubation for 72 h, these were read as per standard antimicrobial susceptibility protocols to determine minimum inhibitory concentrations and CLSI M24-A2 [9] susceptibility breakpoints were used. Findings are described in Supplementary Table 1.

The same antibiotic regimen was continued for three weeks in the hospital. The patient demonstrated adequate recovery with no further cough, sputum, hemoptysis, or chest discomfort. He had a repeat CT chest which uncovered interval improvement of the cavity. He was discharged with oral TMP-SMX and on a follow-up imaging on 07/30/2021, cavitation was still present but it had improved further.

## Discussion

Nocardiosis is an infrequent disorder with an estimated incidence of 500 to 1,000 persons per year in the United States in the 1970s [10]. Nowadays, the prevalence of this disorder appears to be increasing, along with the number of patients at higher risk for this usually opportunistic infection, such as HIV-positive patients and transplant recipients. In an observational study of over a thousand patients, almost two-thirds were found to be immunocompromised, notably due to solid organ or hematopoietic transplant, HIV infection, diabetes mellitus, and notably due to glucocorticoid therapy [11, 12].

Pulmonary nocardiosis is the most frequent clinical manifestation of nocardiosis, accounting for approximately 40% of infections [11, 12], and it is the focus of this case report. The presentation is more commonly subacute or even chronic and is usually suppurative, which may feel counterintuitive to the clinician as the chest imaging may present with cavitary pneumonia.

This case report describes the second documented *Nocardia caishijiensis* infection in a human since its original description in 2003 in Anhui province, China [8]. Given the increasing use of molecular over biochemical analyses for speciation, the *Nocardia* genus has undergone a significant reclassification, resulting in the characterization of new species, such as *Nocardia caishijiensis,* this methodology is also what allowed for the identification of this bacteria in the first documented infection by Rahdar HA et al., [13] in their case series evaluating immunocompromised patients. The different species in the Nocardia genus have been found to have a diverse antimicrobial susceptibility, and appropriate speciation may facilitate the selection of empiric therapy in such patients, although caution should be exercised, given the lack of extensive studies correlating in vitro activity with clinical outcomes.

In terms of management, isolated cutaneous, non-severe mycetoma and mild-to-moderate pulmonary disease are typically treated with oral monotherapy; whereas, severe mycetoma, pulmonary disease, or involvement of multiple organs require intravenous combination therapy with two or three agents. The empiric antimicrobial therapy of choice is TMP-SMX. The other agents typically used include amikacin, imipenem, and third-generation cephalosporins [14–16]. In the past, there was concern regarding high rates of TMP-SMX resistance, however, more recent studies have found that approximately 2.5% of isolates were sulfonamide resistant, and 0.5% of isolates were resistant to trimethoprim, with a reduction in clinical failure on TMP-SMX, further supporting the role of TMP-SMX as the antimicrobial therapy of choice [17]. However, definitive therapy should be adjusted once susceptibility tests are obtained.

Clinical improvement should be expected within three to ten days after initiation of appropriate antimicrobial therapy [16, 18], and lack of improvement after that should prompt the clinician to evaluate for primary drug resistance, inadequate drug penetration into the infectious site, presence of a nocardial abscess and, especially in immunocompromised hosts, the presence of a co-existing infection. The duration of the treatment is variable, however, for serious infections in immunocompromised patients, such as the case described above, treatment should continue for at least one year [19].

## Conclusion

*Nocardia caishijiensis* should be considered in the differential diagnosis of cavitary pneumonia or suspected nocardiosis in the immunocompromised host. The broader implications of this are two-fold. The prevalence of nocardiosis is increasing in line with that of immunocompromised hosts in the modern era; and the emergence of novel nocardial species is likely to continue, given the impact of molecular techniques on nocardial taxonomy. However, further research is required to determine the clinical spectrum of *Nocardia caishijiensis*, as well as the nuances of its diagnosis, and treatment, including antimicrobial therapies and susceptibilities.

## Supplementary Information

**Additional file 1: Supplementary Table 1.**

## Data Availability

All data generated or analyses during this study are included in this published article.

## Abbreviations

TMP-SMX: Trimethoprim-sulfamethoxazole
HIV: Human Immunodeficiency Virus
BAL: Bronchoalveolar lavage
CD4: Cluster of differentiation 4
CT: Computed tomography
