# Supplementary material for: Nocardia caishijiensis infection: a case report and review of the literature
Source: BMC Infect Dis. 2023 Apr 6;23:218. doi: 10.1186/s12879-023-08186-z (PMC10080825; doi:10.1186/s12879-023-08186-z)
Supplement: Supplementary file 1 — Additional file 1: Supplementary Table 1. [file 12879_2023_8186_MOESM1_ESM.pdf]

	MIC (mcg/mL)	*N. caishijiensis*
Trimethoprim-sulfamethoxazole	≤ 1	S
Amoxicillin-clavulanate	> 64/32	R
Ceftriaxone	16	I
Cefepime	> 32	R
Ciprofloxacin	> 4	R
Clarithromycin	> 16	R
Doxycycline	4	I
Imipenem	8	I
Linezolid	2	S
Minocycline	4	I
Moxifloxacin	2	I
Tobramycin	≤ 1	S

MIC: Minimum Inhibitory Concentration; S: Susceptible; I: Intermediate; R: Resistant.
